# Prediction of quality-of-life improvement after total hip arthroplasty: a simplified and internally validated model based on 82,526 total hip arthroplasties from the Swedish Arthroplasty Register

**DOI:** 10.1302/2633-1462.611.BJO-2025-0138.R1

**Published:** 2025-11-21

**Authors:** M. Abdulhadi Alagha, Justin P. Cobb, Alexander D. Liddle, Henrik Malchau, Maziar Mohaddes, Ola Rolfson

**Affiliations:** 1 MSk Lab, Department of Surgery and Cancer, Faculty of Medicine, Imperial College London, London, UK; 2 Harvard Medical School, Boston, USA; 3 Department of Orthopaedics, Hässleholm Hospital, Hässleholm, Sweden; 4 Orthopedics, Faculty of Medicine, Department of Clinical Sciences, Lund University, Lund, Sweden; 5 Department of Orthopaedics, Institute of Clinical Sciences, University of Gothenburg, Gothenburg, Sweden

**Keywords:** Machine learning, Total hip arthroplasty, PROMs, Patient-reported, total hip arthroplasty (THA), visual analogue scale (VAS), EQ-5D, patient-reported outcome measures (PROMs), SAR, Arthroplasty Register, Hip Score, EQ-VAS scores, EuroQol five-dimension questionnaire (EQ-5D), primary elective total hip arthroplasty

## Abstract

**Aims:**

Pain and poor health-related quality of life measures serve as the primary indication for primary elective total hip arthroplasty (THA). It remains challenging to predict whether THA delivers the patient-anticipated improvements. Our study aimed to develop and validate statistical and machine learning prediction models of one-year clinical improvement in patient-reported outcome measures (PROMs) after elective THA.

**Methods:**

We included 82,526 patients with primary elective THAs from the Swedish Arthroplasty Register (SAR) for forecasting one-year improvements in the EuroQol five-dimension questionnaire (EQ-5D) index, EQ-visual analogue scale (VAS), and combined EQ-5D/EQ-VAS scores. Two minimal clinically important difference (MCID) thresholds were applied for the EQ-5D index score based on the approaches of standardized response mean (SRM) of 0.196 and capacity of benefit (CoB) of 0.428. MCID cutoff for the EQ-VAS was set to 7.81. A total of 21 features were used to feed the models. To avoid estimates bias, we eliminated missing data. Model performance was tested using the area under the receiver operating characteristic curve (AUC), and importance of features was identified in the best performing algorithm.

**Results:**

Applying the SRM MCID, approximately two-thirds of patients reported one-year improvements in EQ-5D index (66.3%) and EQ-VAS (69.1%). The improvement rate decreased to 51.7% when we combined improvements in both outcomes. A higher CoB cut-off for EQ-5D index yielded lower rates (~40% for the EQ-5D index and 31.3% for the combined measure). The gradient boosting machine (GBM) consistently outperformed other models by a narrow margin in predicting significant clinical improvements in one-year PROMs and achieved a good to excellent binary discriminative power (AUC range 0.80% to 0.97%). Preoperative PROMs, EQ-5D index, EQ-VAS, and Charnley Hip Score, along with age, collectively contributed to over 80% of the algorithmic power in the ensemble GBM model.

**Conclusion:**

We developed an interpretable machine learning model on a Swedish cohort that may facilitate personalized assessment of meaningful clinical improvement after elective THA.

Cite this article: *Bone Jt Open* 2025;6(11):1504–1514.

## Introduction

Mortality and revision outcomes are the most commonly reported variables following arthroplasty, initially collected by joint registries for the purpose of implant safety surveillance. However, the use of implant failure as endpoint measure is limited and does not reflect the ultimate outcome for patients.^1^ On the other hand, pain and poor health-related quality of life (HRQoL) measures serve as the primary indication for primary elective total hip arthroplasty.^2^ The efforts undertaken by hip joint registries to monitor outcomes related to total hip arthroplasty (THA) culminated in the adoption of HRQoL and disease-specific patient-reported outcome measures (PROMs) collection, which commenced in 2002 in the Swedish Arthroplasty Register (SAR)^2^ and 2009 in the National Joint Registry (NJR) for England and Wales,^3^ and underscore their commitment to address patient experience. While previous studies examined the differences in these measures,^2,4,5^ it remains challenging to predict whether THA delivers the patient-anticipated improvements due to variations in baseline health status, quality of life, and daily activities. Forecasting this exerts a multi-tiered impact, benefitting individuals by facilitating shared decision making, enhancing care delivery at the local level, and informing national policy and economic evaluations.

The EuroQol five-dimension questionnaire (EQ-5D),^6^ despite conceptual differences compared to joint-specific PROMs, such as the Oxford Hip Score (OHS) and Harris Hip Score (HHS), uses standardized questions to comprehensively assess individuals’ physical and mental wellbeing, and pain and functional levels across five domains: mobility, self-care, usual activities, pain/discomfort, and anxiety/depression. The EQ-5D index represents a global measure of HRQoL, calculated based on specific weight value set, whereas a visual analogue scale (EQ-VAS) is separately recorded for general health.^7^ This tool was shown to have concurrent validity, acceptable ceiling effects, and high discriminatory power in several patient groups including THA.^8-11^

A recent systematic review sought to discern the predictors of EQ-5D and EQ-VAS changes among individual patients undergoing THA with findings indicative of limited or inconclusive evidence in this regard.^12^ Two studies constructed machine learning (ML) models to forecast changes in PROMs (EQ-5D index and EQ-VAS) following hip arthroplasty; however, despite achieving a good binary discriminative power, they were constrained either by their limited and non-representative sample size or the absence of relevant clinical data.^13,14^ Clinically, forecasting PROMs outcomes supports more individualized risk communication and managing of waiting lists by providing patient-specific outcome predictions, thereby moving beyond reliance on broad population-level success rates typically cited as 90% to 95%.^15,16^

The aim of this study was to develop ML models that predict meaningful improvements in one-year HRQoL and compare their predictive ability to traditional statistical linear models, using a large dataset from the SAR.

## Methods

The SAR dataset (n = 442,546) was used for model derivation and internal validation for the prediction of improvements in one-year EQ-5D and EQ-VAS in patients operated between January 2000 and December 2018. The study focused on adult patients undergoing elective primary hip arthroplasty, and we thus excluded cases with a diagnosis of trauma, tumour, and non-osteoarthritic degenerative conditions. Release of data for the purposes of this study was approved by the Swedish Arthroplasty Register (SAR) steering group on 12 December 2019 (Reference no: 804 to 17), with ethical approval provided via the University of Gothenburg.

Data related to primary hip arthroplasty are collected for all nationwide public and private hospitals in Sweden and reported to the register with an estimated completeness of 98%.^17^ Our inclusion period started in 2000 due to the absence of systematic inclusion of BMI and the American Society of Anesthesiologists (ASA) grade^18^ in earlier records. Between 2000 and 2008, both ASA and BMI exhibited a low level of completeness (> 50% missing data), and thus non-missing data for these variables were retained in the analysis. The extract contained patients’ demographic characteristics and operative and component-level data, and the SAR is periodically synchronized with the Tax Office and National Patient Register (NPR),^19^ providing information on mortality, causes, and time to event. We examined all variables for missing data, and records with missing values exceeding 60% were excluded under the assumption that the missing data occurred at random (n = 154,595 THAs; Table I). Observations characterized by the absence of recorded preoperative and postoperative PROMs data (Charnley Hip Score,^20^ EQ-5D index, EQ-VAS) were omitted from the dataset, resulting in a final dataset of 82,526 THAs. In addition to preoperative PROMs, the cleaned dataset included 21 variables (Table II): six patient variables (age, sex, BMI, ASA, side of operation, and the type of hospital); four surgical variables (diagnosis group, incision type, prosthesis group, and cement type); and 11 implant-specific variables, which were each subdivided into six cup variables and five femoral variables (fixation, resurfacing, articulation, modular or monoblock, and implant size, in addition to the sixth cup-related variable 'modification'). Potential correlations among predictors were examined, and any highly correlated variables were excluded to address multicollinearity and ensure model robustness. Each variable is described in more detail in Suppmentary Material 1.

**Table I.** Baseline characteristics of the study population in the Swedish Arthroplasty Register after data cleaning.

Variable	Data
Operations, n	154,595
Mean age at surgery, yrs (SD)	67.9 (10.6)
Male sex, n	66,868 (43.3)
**BMI, kg/** ^**2**^ **, n (%)**	
Underweight	1,191 (0.8)
Normal	48,037 (31.1)
Overweight	66,408 (43.0)
Obesity class I	29,757 (19.2)
Obesity class II	7,647 (4.9)
Obesity class III	1,555 (1.0)
**ASA grade, n (%)**	
I	36,826 (23.8)
II	92,076 (59.6)
III +	25,693 (16.6)
Right side, n (%)	70,000 (45.3)
**Unit type, n (%)**	
University hospital	12,244 (7.9)
County hospital	47,052 (30.4)
Rural hospital	60,254 (39.0)
Private hospital	35,045 (22.7)
**Preoperative diagnosis, n (%)**	
Primary arthrosis	1,412 (91.5)
Inflammatory	1,962 (1.3)
Childhood diagnosis	3,256 (2.1)
Idiopathic necrosis	3,741 (2.4)
Secondary arthrosis	4,097 (2.7)
Other	127 (0.1)
**Incision type, n (%)**	
Posterior	83,146 (53.8)
Direct lateral	70,924 (45.9)
Direct anterior	324 (0.2)
Trochanteric	173 (0.1)
Other	12 (0.0)
**Prosthesis group, n (%)**	
Cemented	95,490 (61.8)
Cementless	33,479 (21.7)
Hybrid	5,030 (3.3)
Reverse hybrid	19,528 (12.6)
Resurfacing	1,068 (0.7)
**Cement type, n (%)**	
High viscosity with antibiotic	55,195 (35.7)
Low viscosity with antibiotic	876 (0.6)
High viscosity without antibiotic	58,915 (38.1)
Low viscosity without antibiotic	11 (0.0)
Cementless, hybrid or resurfacing	39,577 (25.6)
**Cup fixation, n (%)**	
Cemented	115,020 (74.4)
Cementless	39,575 (25.6)
Cup resurfacing	1,528 (1.0)
Cup modulaity (Monoblock)	115,818 (74.9)
**Cup articulation, n (%)**	
Metal (standard)	235 (0.2)
Metal (resurfacing)	1,531 (1.0)
Ceramic	637 (0.4)
Dual-mobility (monoblock)	1,278 (0.8)
Dual-mobility (modular)	5 (0.0)
Poly (standard)	44,116 (28.5)
Poly (x-link)	106,628 (69.0)
Unclear	165 (0.1)
**Cup modification, n (%)**	
Standard	100,836 (65.2)
Liped	12,319 (8.0)
Dual articular	1,146 (0.7)
Constrained	10 (0.0)
Unclear	40,284 (26.1)
Mean cup inner diameter (SD)	31.49 (3.05)
**Femoral component fixation, n (%)**	
Cemented	101,585 (65.7)
Cementless	53,010 (34.3)
Femoral component resurfacing	1,068 (0.7)
Femoral component modularity (Modular)	153,466 (99.3)
**Femoral component articulation, n (%)**	
Metal	127,730 (82.6)
Ceramic	25,766 (16.7)
Unclear	1,099 (0.7)
Mean femoral head size, mm (SD)	31.49 (3.03)

ASA, American Society of Anesthesiologists.

**Table II.** Sources of variables used for model development.

Factor	Swedish Arthroplasty Register
Patient factors	Age
	Sex
	BMI
	ASA grade
	Side of operation
	Hospital type
Surgical factors	Operation type
	Diagnosis group
	Incision type
	Prothesis group
	Cement type
Cup implant factors	Fixation
	Resurfacing
	Articulation
	Modular or Monoblock
	Inner diameter
	Modification
Femoral implant factors	Fixation
	Femoral component resurfacing
	Articulation
	Head size
Preoperative PROMs	EQ-5D
	EQ-VAS
	Charnley Hip Score
One-year outcomes	EQ-5D, SRM threshold
	EQ-5D, paired threshold
	EQ-VAS
	Combined EQ-5D (SRM)/EQ-VAS
	Combined EQ-5D (paired)/EQ-VAS

ASA, American Society of Anesthesiologists; EQ-5D, EuroQol five-dimension questionnaire; EQ-VAS, EuroQol visual analogue scale; PROMs, patient-reported outcome measures; SRM, standardized response mean.

This study is in two parts: the first uses the SAR dataset to develop and internally validate the ML algorithms in predicting one-year clinically important improvements in EQ-5D index, EQ-VAS, and combined EQ-5D/EQ-VAS scores. This is followed by a comparison of the essential features influencing these predictions in the best performing ML model.

### Outcome of interest

The primary outcome measure in the SAR dataset was one-year improvements in the EQ-5D index, EQ-VAS, and combined EQ-5D/EQ-VAS scores, as set by the minimal clinically important difference (MCID) thresholds described below.

There are two variations of the EQ-5D questionnaire: a three-level (3L) version and the newer five-level (5L) release. EQ-5D-5L adds two additional layers of severity response levels in attempts to address the ceiling effects, the low sensitivity, and enhance the discriminatory power of the 3L version.^10,21,22^ The SAR adopted PROMs data collection using the 3L version up until 2017, at which point it transitioned to the 5L version. Eneqvist et al^23^ compared the two versions in western Sweden in terms of redistribution of responses, ceiling and floor effects, and correlations of EQ-VAS and EQ-5D indices. The authors reported a good agreement for EQ-VAS (Spearman’s rank correlation coefficient, *r* = 0.71 preoperatively; Spearman *r* = 0.87 postoperatively) while the 5L questionnaire had 7% lower ceiling effects when compared to 3L.

**EQ-5D index, EQ-VAS, and combined EQ-5D/EQ-VAS:** The differences between the preoperative and one-year postoperative EQ-5D index and EQ-VAS were calculated and assigned a binary outcome 'improved' and 'not improved' based on set MCID thresholds. Additionally, the overall combined improvement of the EQ-5D index and EQ-VAS was assessed. Table II highlights the study outcomes.

**MCID:** MCID refers to the smallest improvement deemed significant and meaningful by a patient and varies by patient cohort, conditions, and instrument.^24,25^ Several methods exist to calculate this, including: paired-data specific; anchor-based; and distribution-based MCIDs. The choice of which value to select depends on the nature of the study and the research question. Previous studies selected MCID for the EQ-5D index using either the Walters et al’*s*^26^ anchor-based MCID of 0.074, or the statistical effect size of Cohen’s MCID of 0.2.^27^ However, these thresholds are uncertain and not specific to hip osteoarthritis or THA treatment.^12^

More recently, Kang^28^ measured the internal responsiveness of the EQ-5D index for hip arthroplasty using NHS PROMs data between 2009 and 2015 (n = 181,424) and a variety of methods including univariate and multivariate standardized response mean (SRM), standardized effect size (SES), and paired data-specific MCID while adjusting for baseline covariates such as age, sex, and comorbidities. They reported EQ-5D index MCID values of 0.196 for the SRM-applied method with a desired effect size equivalent to Cohen’s medium effect (0.5) - a value exceeding 0.196 signifies a meaningful improvement from a clinical perspective. The adjusted multivariate capacity of benefit score, using the paired-data method with applied SRM as a desired effect size, was measured to be 0.428. The latter represents the magnitude of improvement that would be considered of substantial clinical benefit.

In terms of EQ-VAS, a recent study applied ML models and derived a MCID value of 7.81 using the change difference method in a sample of 1,843 THAs with an area under the receiver operating characteristic curve (AUC) of 0.84,^14^ which is within the range of a Danish study, which used multiple anchor-based approaches and reported a calculated hip minimal MCID range between 5 and 23.^29^Table III highlights the designated MCIDs in this study.

**Table III.** Minimal clinically important difference thresholds used per outcome.

Outcome	MCID
EQ-5D, SRM	0.196
EQ-5D, capacity of benefit	0.428
EQ-VAS	7.81

EQ-5D, EuroQol five-dimension questionnaire; EQ-VAS, EuroQol visual analogue scale; MCID, minimal clinically important difference; SRM, standardized response mean.

### Model development, training, and validations

Six ML algorithms were developed, namely random forest (RF), gradient boosting machine (GBM), penalized logistic regression (with Lasso and Ridge penalty), and classification tree (with and without pruning), and then compared to traditional statistical model logistic regression to predict the improvement in one-year PROMs. The SAR derivation cohort of 82,526 observations was used for model development and internal validation. The data extract was randomly split into a training dataset (n = 66,020, 80% of the patients) and an internal validation cohort (n = 16,506, 20% of the patients). The training cohort was used to train the ML models and to adjust their hyperparameters via cross-validation whereas the validation cohort was used to assess the models’ performance on unseen data. Model performance was evaluated and compared in terms of AUC in each dataset. The top performing ML model was used to identify the key features driving its performance.

### Statistical analysis

Descriptive statistics including mean, median, and percentage were used to describe the rates of one-year PROMs changes, and across various prosthesis groups. All mathematical modelling in the SAR dataset was carried out using R statistical computing environment version 4.3.0 (R: A language and environment for statistical computing). R packages ‘gbm’ v. 2.1.8.1, ‘glmnet’ v. 4.1.7, ‘tree’ v. 1.0.43, ‘rpart’ v. 4.7.19, and ‘randomForest’ v. 4.7.1.1, were used for dichotomous classification prediction (R Foundation for Statistical Computing, Austria).

## Results

### Summary statistics of outcomes

Descriptive figures related to the changes in one-year PROMs across the different thresholds are presented in Table IV. Using the SRM cut-off, approximately two-thirds of patients reported a one-year improvement in each HRQoL domain (EQ-5D index 66.3%, EQ-VAS 69.1%). This decreased to 51.7% when we combined improvements between the two outcomes. Using a higher cutoff value for EQ-5D index yielded lower rates (~40% for the EQ-5D index and 31.3% for the combined one). Table V shows the percentages of improvements per prosthesis group across the five outcomes. Overall, hip resurfacing or uncemented and reverse hybrid fixations were variably observed to have the highest rates of improvements in one-year PROMs.

**Table IV.** Analysis of study outcome rates in the Swedish Arthroplasty Register extract.

Outcome at one-year	Derivation cohort (n = 82,526), n %
EQ-5D index, SRM threshold improved)	54,734 (66.3)
EQ-5D index, CoB threshold (improved)	32,890 (39.9)
EQ-VAS (improved)	57,064 (69.1)
Combined SRM EQ-5D SRM and EQ-VAS (improved)	42,653 (51.7)
Combined EQ-5D CoB and EQ-VAS (improved)	25,849 (31.3)

CoB, capacity of benefit; EQ-5D, EuroQol five-dimension questionnaire; EQ-VAS, EuroQol visual analogue scale; SRM, standardized response mean.

**Table V.** Analysis of study outcome rates in the Swedish Arthroplasty Register extract per prosthesis group.

Prosthesis group	EQ-5D CoB improvement, n (%)	EQ-5D SRM improvement, n (%)	EQ-VAS improvement, n (%)	Combined EQ-5D CoB + EQ-VAS, n (%)	Combined EQ-5D SRM + EQ-VAS, n (%)
Cemented	21,289 (39.2)	34,750 (64.0)	36,834 (67.8)	16,440 (30.2)	26,789 (49.3)
Uncemented	6,088 (40.4)	10,777 (71.6)	10,807 (71.8)	4,956 (32.9)	8,574 (56.9)
Hybrid	850 (45.3)	1,335 (71.2)	1,213 (64.7)	630 (33.6)	979 (52.2)
Reverse hybrid	4,442 (41.9)	7,326 (69.2)	7,631 (72.0)	3,623 (34.2)	5,848 (55.2)
Resurfacing	221 (29.0)	546 (71.6)	579 (75.9)	200 (26.2)	463 (60.7)

CoB, capacity of benefit; EQ-5D, EuroQol five-dimension questionnaire; EQ-VAS, EuroQol visual analogue scale; SRM, standardized response mean.

### Model development, training, and validation

**Fig. 1:**
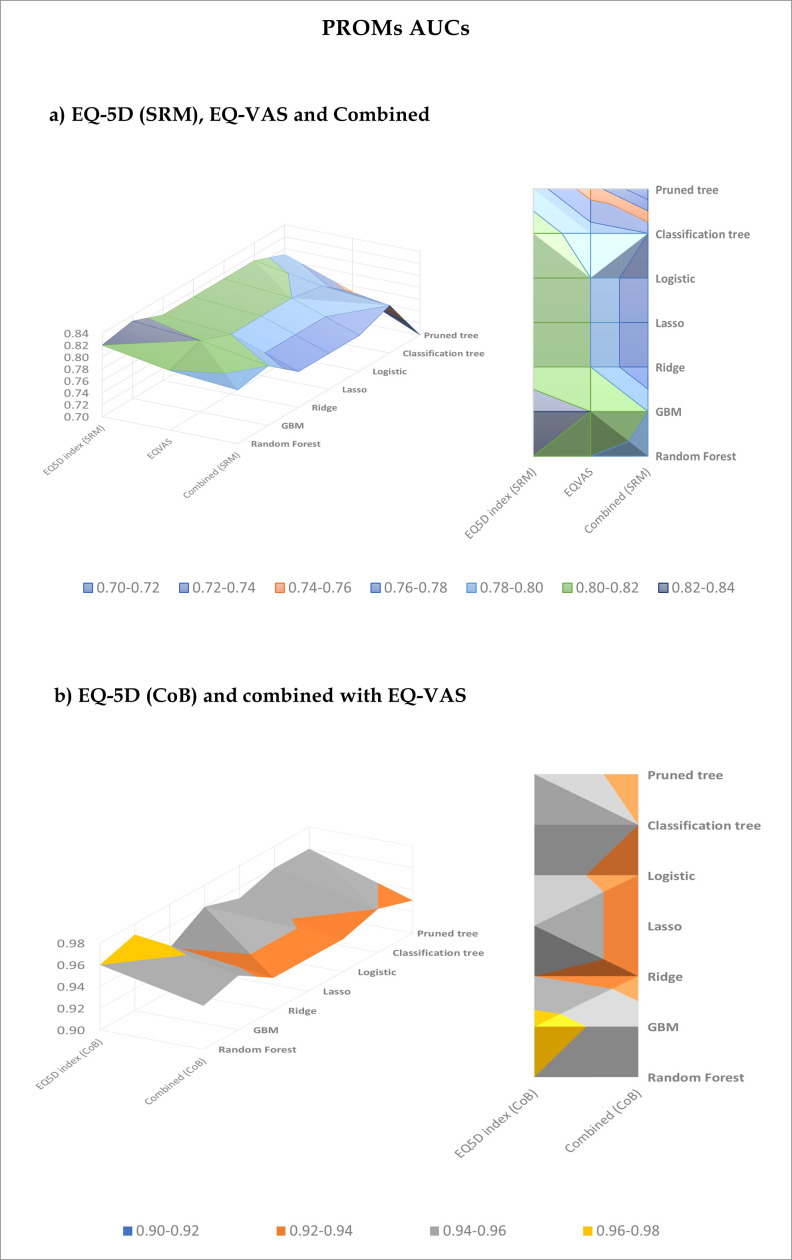
3D surface and contour visualization of patient-reported outcome measures (PROMs) predictive models’ area under the curve (AUC) values in the Swedish Arthroplasty Register. X axes represent the minimal clinically important difference thresholds used; Y axes show the different algorithms applied. CoB, capacity of benefit; EQ-5D, EuroQol five-dimension questionnaire; GBM, gradient boosting machine; SRM, standardized response mean; VAS, visual analogue scale.

GBM was consistently the best performing ML algorithm in predicting significant clinical improvements in one-year PROMs (AUCs range 0.80% to 0.97%), outperforming the other models by a narrow margin (Table VI*;*Figure 1).

**Table VI.** Patient-reported outcome measures model performance per minimal clinically important difference in the Swedish Arthroplasty Register.

Outcome	Model performance, AUC % (95% CI)						
	Random forest	Gradient boosting machine	Ridge regression	Lasso regression	Logistic regression	Classification tree	Classification tree with pruning
EQ-5D index, SRM	0.82 (0.81 to 0.83)	0.83 (0.82 to 0.84)		0.81 (0.80 to 0.82)			0.79 (0.78 to 0.79)
EQ-5D index, CoB	0.96 (0.95 to 0.96)	0.97 (0.96 to 0.97)	0.94 (0.94 to 0.95)	0.96 (0.95 to 0.96)	0.95 (0.95 to 0.96)	0.96 (0.96 to 0.96)	0.96 (0.95 to 0.96)
EQ-VAS	0.80 (0.79 to 0.81)	0.82 (0.81 to 0.82)	0.80 (0.80 to 0.81)	0.80 (0.80 to 0.81)	0.80 (0.80 to 0.81)	0.79 (0.78 to 0.80)	0.75 (0.74 to 0.76)
Combined, CoB	0.94 (0.94 to 0.95)	0.95 (0.95 to 0.95)	0.93 (0.93 to 0.93)	0.93 (0.93 to 0.93)	0.93 (0.93 to 0.94)	0.94 (0.94 to 0.94)	0.93 (0.93 to 0.94)
Combined, SRM	0.79 (0.78 to 0.80)	0.80 (0.79 to 0.81)	0.76 (0.75 to 0.76)	0.76 (0.75 to 0.77)	0.76 (0.75 to 0.77)	0.78 (0.77 to 0.78)	0.70 (0.69 to 0.71)

AUC, area under the receiver operating characteristic curve; CoB, capacity of benefit; EQ-5D, EuroQol five-dimension questionnaire; EQ-VAS, EuroQol visual analogue scale; SRM, standardized response mean.

### Feature importance and response analysis

As the GBM was the top-performing model, we proceeded to examine the responses and the significance of the features identified by this model. While these features provide insights into the model internal decision-making process, caution is strongly advised when interpreting the results since feature importance is not subject to inferential testing and does not establish statistical significance.

**Fig. 2:**
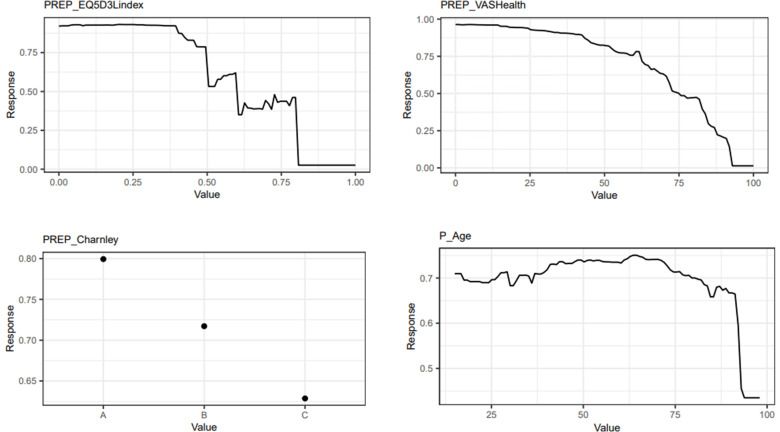
The three most important features for the gradient boosting machine predictive models. The top two are the number one feature for EuroQol five-dimension three-level questionnaire (EQ-5D-3L) index and EQ-visual analogue scale (VAS) outcomes, respectively. Preoperative Charnley Hip Score and age were the second and third ranked features, respectively.

Three features consistently emerged as key drivers for the predictions of changes in the EQ-5D index (SRM and CoB thresholds), and EQ-VAS, and these include, in ranked sequence: preoperative EQ-5D index (importance: SRM 73.1%; and 95.8% for CoB) and EQ-VAS (importance 72.2%), respectively; preoperative Charnley Hip Score (importance: SRM 9.0%; CoB 0.9%; and 6.3% for EQ-VAS), and age (importance: SRM 3.3%; CoB 0.6%; and 4.2% for EQ-VAS) (Figure 2).

A lower preoperative PROMs score appeared to be linked with better postoperative improvements. On the other hand, age below 75 years tended to have a similar improvement in HRQoL but this sharply declined with increasing age. Given the importance of these three features in driving the EQ-5D CoB threshold model (totalling 97.3%), we excluded further feature analysis for the remaining 2.7%. Supplementary Figure a identifies the remaining six key features driving the predictions for changes in EQ-5D index (SRM threshold) and EQ-VAS.

There appeared to be a positive correlation between two arms of the EQ-5D (index and VAS), wherein higher preoperative values in one arm seemed to improve one-year PROMs in the other. In both outcomes, the extremes of ASA grades were identified by the model to augment PROMs improvement at one year, while obesity classes I to III were observed to have less pronounced improvements. Hip resurfacing and uncemented and reverse hybrid fixation were shown by the model to have the highest response effect on one-year PROMs. Care delivered at private units was identified to have better improvements in one-year EQ-VAS scores, but not EQ-5D. There were similar improvement levels among all surgical indications studied, except for the 'other' group.

## Discussion

This study has shown that predicting one-year improvements in HRQoL following primary elective hip arthroplasty using ML algorithms achieved a good to excellent binary discriminative power and slightly outperformed conventional statistical predictive models. This is the first study to incorporate clinical and implant-specific registry data to generate personalized stratification models, which if applied in clinical practice, may improve the shared decision-making process.

The descriptive statistics allude to the fact that approximately two-thirds of individuals reach a significant clinical improvement at one-year postoperatively in the health utility index (66.3%) and general health (69.1%), while only 40% reached substantial meaningful improvement in their EQ-5D index score at one year. Our figures are comparable to previous studies,^13,14,30^ reporting 53.4%^28^ to 58%^14^ and 44.5%^13^ to 66.3%^14^ of patients reaching EQ-5D and EQ-VAS MCIDs, respectively, using similar cutoff levels. This denotes the population-level consistency and generalizability of our dataset.

With regards to EQ-VAS, our ML models showed strong classification performance in identifying patients with meaningful clinical improvement following THA after one year, equating 0.82 AUC for EQ-VAS using the GBM model. The model found that lower preoperative VAS health and Charnley Hip Score categories and age less than 75 years to be predictor factors of general health improvement, totalling around 83% of feature importance. Huber et al^13^ used the NHS PROMs hip dataset (n = 62,429) between 2015 and 2017 to predict changes in EQ-VAS with a minimal important difference (MID) threshold of 11 points. The authors reported an AUC value of 0.87 for the GBM model outperforming linear models and highlighted that preoperative VAS scores and limping were the key predictors behind their algorithms. While it aligns with our findings, the lack of clinical and implant-specific data and the use of half a SD of baseline preoperative-VAS score as a MID threshold, rather than the standardized response mean or paired data-specific MCID, may have limited the clinical applicability and generalizability of their models.

In terms of health utility (EQ-5D) index, the GBM models reached AUC values of 0.83 and 0.97 in forecasting significant and substantial clinical improvements at one year. Similar to EQ-VAS, this predictive performance heavily relied on the three aforementioned features, with the caveat of preoperative EQ-5D index score rather than EQ-VAS, reaching 85.4% to 97.3% feature importance for significant and substantial improvements, respectively. This agrees with a recent German study,^14^ which provided instrumental calculations for the MCID of 7.83 for EQ-VAS which was implemented in this study. The research team analyzed 1,843 THA observations collected from nine hospitals in Germany with AUC values of 0.81 for the EQ-5D index and 0.84 for the EQ-VAS, echoing prior results. However, the authors acknowledged the need for a larger and representative sample size to enhance the models’ external validity.

When we predicted overall combined improvement of EQ-5D and EQ-VAS, our predictive accuracy marginally declined by approximately 2%, permitting further differentiation of patients likely to have higher HRQoL improvements postoperatively. While the majority of GBM’s predictive ability stemmed from these three features, our models highlighted additional factors possibly contributing to the predictions. Of particular importance, obese patients were noted to exhibit lower improvement scores, while procedures such as hip resurfacing and uncemented femoral fixation displayed the highest response effect. The latter observations while were also evident in our descriptive figures, might be attributed to age-related selection bias. Previous studies tended to exclude BMI as a predictor of functional improvements.^31-35^ However, it is important to note that most of these studies focused on condition-specific PROMs rather than HRQoL. In contrast, while some authors have attributed the increased safety portfolio of hip resurfacing arthroplasty (HRA) to patient selection bias,^36,37^ there is evidence to support superior functional outcomes in HRA.^38-44^

While the limitations related to the retrospective analysis of prospective data collected in a single national dataset remain, the alignment with prior research findings, in terms of descriptive statistics and AUC values, may indicate a degree of population-level consistency. The lack of control groups prevented the construct of patient trajectories without THA, meaning the study cannot discern whether the limited improvements postoperatively were solely attributed to the procedure itself or if THA prevented a substantial deterioration in HRQoL. Similarly, the lack of randomization and blinding in observational studies render them susceptible to systemic bias relating to patient selection. For instance, over the time course of the registry data being reported, HRA was only offered by a small minority of surgeons and to a small group of patients. Moreover, it is important to acknowledge that the PROMs only offer a snapshot assessment of health-related quality of life on a single day, potentially not accounting for temporal variations. The large amount of missing data, particularly incomplete ASA grades in SAR prior to 2008, and our assumption of missing data being completely at random by eliminating missing observations rather than imputing variables, may have introduced bias to the findings. However, imputation introduces uncertainty and may result in biased estimates, which our methodology aimed to avoid.^45,46^ Future studies should explore multiple imputation techniques and conduct sensitivity analyses to assess the impact of different missing data assumptions on model performance and generalizability. Additionally, we acknowledge the possible dependency between baseline EQ-5D and MCID-defined outcomes, however, we included baseline EQ-5D in the models to reflect routine clinical practice, where it informs preoperative decision making. Similarly, while standardized cut-off MCID levels were carefully employed, different thresholds may yield different results, whereas the choice of a binary outcome (improvement or no improvement) may have restricted the models’ ability in forecasting the extent of changes. Similarly, our models were compared using the AUC metric, which is regarded as a gold standard in evaluating classifier performance across a range of thresholds and allows comparisons of multiple ML models. Nonetheless, the AUC-receiver operating characteristic curve-based metric neglects to address the balance of true positives and true negatives, which may affect the accuracy and generalizability of the predictive model which alternative measures seek to address. While the need to specify a threshold in other approaches is known to introduce variations in clinical practice, future studies should aim to adhere to the TRIPOD-AI reporting guidelines.^47^ Furthermore, one ought to consider the potential for systematic bias related to implant selection, where surgeons or units may limit the choice of implants for THA. This is an inherent limitation of observational and registry data and may limit the generalizability and interpretability of the model. Likewise, it is noteworthy that patients who underwent previous THA, i.e. bilateral THA, were not excluded. Future studies should consider a multilevel model incorporating random effects and repeated measures.^48^ Finally, although the features identified by the best performing model provided insights into its ensemble internal decision-making process, this approach is not subject to inferential testing assumptions and therefore inherently limited in evaluating associations. Future studies should seek to externally validate our algorithms on other datasets, such as the NJR for England and Wales, as well as forecast improvements using condition-specific PROMs such as the OHS or HHS, which are not captured by the SAR. Prospective clinical trials are needed to assess the applicability and accuracy of these predictions using a mix of PROMs in clinical settings.

In conclusion, this is the first study to incorporate patient, surgical, and implant-specific factors to forecast one-year improvements in HRQoL measures following primary elective THA. The internally validated ML models for each outcome marginally outperformed the logistic regression model and showed good to excellent discriminative power in predicting significant and substantial clinical improvements, respectively. Three features appeared to account for over 80% of the algorithmic power in the ensemble GBM model including: preoperative baseline outcome score; preoperative Charnley Hip Score; and age. Further research is needed to investigate the optimal set of PROMs, such as EQ-5D, OHS, and HHS, that aid surgical decision-making.

**Take home message**

- This is the first study to develop machine learning models to forecast the improvements in qualiity of life measures after elective total hip arthroplasty using a Swedish cohort.

- Clinically, this study supports more individualized risk communication and manage waiting lists by providing patient-specific outcome predictions, thereby moving beyond reliance on broad population-level success rates typically cited as 90 to 95%.

- The gradient boosting machine (GBM) algorithm, using two minimal clinically important difference cutoffs (standardized response mean, and capacity of benefit), consistently outperformed other models by a narrow margin and achieved a good to excellent binary discriminative power (area under the curve range 0.80% to 0.97%).

- Preoperative patient-reported outcome measures (PROMs), EuroQol five-dimension index, EuroQol visual analogue scale, and Charnley classification, along with age, collectively contributed to over 80% of the algorithmic power in the ensemble GBM model.

- Normal to low BMI, hip resurfacing arthroplasty, and uncemented femoral fixation exerted a comparatively lower impact on the algorithms, suggesting their potential to have highest response effect on one-year PROMs. However, it is important to note that our approach is not subject to inferential testing assumptions and therefore inherently limited in evaluating associations.

## Data Availability

The data that support the findings for this study are available to other researchers from the Swedish Arthroplasty Register steering committee upon reasonable request.
